# Crescent Antennas as Sensors: Case of Sensing Brain Pathology

**DOI:** 10.3390/s24041305

**Published:** 2024-02-18

**Authors:** Usman Anwar, Tughrul Arslan, Peter Lomax

**Affiliations:** 1 School of Engineering, The University of Edinburgh, Edinburgh EH9 3FF, UK; peter.lomax@ed.ac.uk; 2 Advanced Care Research Centre, The University of Edinburgh, Edinburgh EH16 4UX, UK

**Keywords:** brain atrophy, brain sensing, crescent sensors, dementia, flexible sensors, radio frequency, stroke, ultra-wideband

## Abstract

Microstrip crescent antennas offer compactness, conformability, low profile, high sensitivity, multi-band operability, cost-effectiveness and ease of fabrication in contrast to bulky, rigid horn, helical and Vivaldi antennas. This work presents crescent sensors for monitoring brain pathology associated with stroke and atrophy. Single- and multi-element crescent sensors are designed and validated by software simulations. The fabricated sensors are integrated with glasses and experimentally evaluated using a realistic brain phantom. The performance of the sensors is compared in terms of peak gain, directivity, radiation performance, flexibility and detection capability. The crescent sensors can detect the pathologies through the monitoring of backscattered electromagnetic signals that are triggered by dielectric variations in the affected tissues. The proposed sensors can effectively detect stroke and brain atrophy targets with a volume of 25 mm^3^ and 56 mm^3^, respectively. The safety of the sensors is examined through the evaluation of Specific Absorption Rate (peak SAR < 1.25 W/Kg, 100 mW), temperature increase within brain tissues (max: 0.155 °C, min: 0.115 °C) and electric field analysis. The results suggest that the crescent sensors can provide a flexible, portable and non-invasive solution to monitor degenerative brain pathology.

## 1. Introduction

Neurodegeneration refers to the progressive degeneration of the structure and function of neurons in the nervous system [1]. The neurodegenerative process begins with mild cognitive impairment, which leads to cognitive decline, dementia and Alzheimer’s disease [2]. The accumulation of misfolded protein results in the formation of protein plaques, which disrupt normal cellular processes in the case of Alzheimer’s disease. These protein aggregates trigger a cascade of neurodegenerative events that lead to neuronal inflammation. In some cases, this results in neuronal dysfunction and the synaptic loss of communication between neurons [3]. In the advanced stages of neurodegeneration, the loss of neurons leads to the gradual shrinking of specific brain regions which is mostly irreversible. Therefore, early diagnosis and treatment are crucial in slowing down neurodegenerative diseases.

Wearable radio frequency (RF) sensors can monitor various physiological and pathophysiological indicators of neurodegenerative conditions, allowing for early intervention and potentially decelerating disease progression. Traditional diagnostics are mostly periodic and are not suitable for continuous monitoring. Wearable sensors offer real-time, non-invasive, unobtrusive and continuous monitoring, which can capture objective data to supplement clinical assessments. The data can be analyzed on-edge or transmitted to the cloud for remote monitoring and neurodiagnostics. This is especially useful for patients with mobility limitations and can lead to robust diagnostics with effective intervention. The recent advancements in wearable sensing include wearable, portable, implantable and ingestible sensors for remote monitoring [4]. Wearable sensors provide non-invasive sensing which allows minimal intervention for neurodiagnostic sensing. Ultra-wideband (UWB) frequency can non-invasively penetrate the brain with a low-profile sensor configuration. UWB sensors can integrate with a cap, headband or glasses to scan the brain for degeneration.

Wearable RF sensing techniques have been the focus of recent research. Several technologies have been presented recently for brain tumour [5], haemorrhagic stroke [6], lateral ventricle enlargement [7] and brain cancer [8]. Most of these techniques required at least six or more antenna elements to realize complete brain coverage. These techniques lacked dynamic device configuration, power optimization and portability. Moreover, the RF sensors presented in these studies were able to detect a single anomaly. To overcome these challenges, the proposed work presents multimodal RF sensors, which can detect and monitor multiple pathologies and their progression. The proposed sensing technique can operate with two RF sensors integrated with either side of the glasses to scan the frontal, temporal and parietal regions of the brain. Ischemic stroke and brain atrophy are the major pathologies associated with dementia. The performance of the proposed crescent sensors is validated with both computational and experimental models. The crescent sensors can detect these anomalies through the monitoring of dielectric variation between healthy and diseased tissues. This study presents non-invasive, low-cost and miniaturized crescent sensors with both rigid and flexible structures. The minimalistic sensor designs offer a low profile, improved bandwidth, high directivity and improved sensitivity as compared to the literature. The integration of sensors with glasses ensures low-complexity and wearability in contrast to the head-mounted sensor systems presented in the literature. The choice of glasses in this work offers a minimalistic design to examine frontal, parietal and temporal regions of the brain. The proposed crescent sensors comply with the wearable health and safety standards, which makes them suitable for pre-clinical trials.

The paper is organized as follows: Section 2 discusses the prospects of neurodiagnostics with radio frequency sensors. Section 3 presents the design and performance evaluation of single-element crescent sensors. The performance is assessed through software simulations with a focus on reflection characteristics, gain, directivity and radiation performance of the sensors. Section 4 provides a detailed analysis of the design and software modelling of multi-element crescent sensors. The performance of multi-element sensors is evaluated through reflection features, peak gain, directivity and radiation performance. The design and performance of a flexible slotted crescent sensor is discussed in Section 5. Section 6 presents the results of the near-brain simulations along with the near-field evaluation to assess health and safety features. This includes the measurement of specific absorption rate (SAR), thermal characterization and electric field analysis. Experimental validation using a realistic brain phantom with stroke and brain atrophy pathology is presented in Section 7. The measured results are taken from all the sensors and analysed for both stroke and brain atrophy targets. Section 8 presents a discussion on fabrication materials and techniques of wearable sensors from the literature in comparison to the proposed crescent sensors. Section 9 presents a comparative analysis with state-of-the-art brain sensing technologies. The applications of crescent sensors, including opportunities and challenges for wearable brain sensing, are discussed in Section 10. The paper concludes with a brief discussion of the future scope in Section 11.

## 2. Neurodiagnostics with Radio Frequency Sensors

Radio frequency sensing is an evolving research area with numerous potential applications in neurodiagnostics, cognitive assessment, clinical diagnostics and brain-computer interface (BCI) development. Although traditional medical technologies are immensely valuable in diagnosis and treatment planning, they require extensive medical supervision and are not easily accessible to the patients. Prolonged exposure to ionizing radiation in the case of X-ray and computed tomography (CT) carries a risk of radiation-induced cancer [9]. Magnetic resonance imaging (MRI) and positron emission tomography (PET) scans are expensive to acquire, maintain and operate [10]. The limited temporal resolution, motion artifacts and patient discomfort also limit the efficacy of these conventional technologies.

Radio frequency (RF) sensors provide a non-invasive, wearable and unobtrusive way to detect and monitor different brain diseases. RF sensing involves the use of radio frequency signals to detect these anomalies without direct contact with the scalp. RF signals can penetrate tissues, skulls and white and grey matter to allow the monitoring of brain activity and degeneration. RF sensing offers improved spatial resolution compared to traditional electroencephalography (EEG) and can provide a more comprehensive view of the brain compared to surface-based methods.

**Figure 1:**
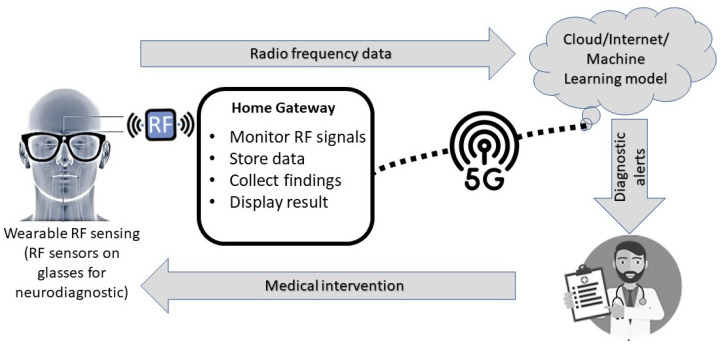
Remote brain sensing and neurodiagnostics with wearable radio frequency (RF) sensors.

RF sensing can be utilized to proactively monitor dementia, which is a progressive neurodegenerative disorder that affects cognitive functions. The neurodegenerative pathology associated with dementia and Alzheimer’s disease includes atrophy [11], plaques [12], tau-tangles [13] and protein deposits [14]. The blood density variation can be measured with RF sensing, which is an effective biomarker for ischemic stroke in vascular dementia [15]. The RF sensing data can be processed for the image reconstruction of structural and functional pathological changes associated with dementia [16,17]. The ongoing advancements in medical imaging technology include hybrid imaging and diagnostics with integrated machine-learning models for the classification of RF sensing data and scanned brain images [18]. This will enable healthcare specialists to remotely detect and monitor dementia progression. The early diagnosis of progressive dementia allows for better management and treatment. Proactive remote monitoring, diagnosis and intervention can help slow down the progression with cognitive rehabilitation and individualized dementia management. Wearable sensing provides several advantages in terms of continuous monitoring, portability, non-invasiveness, robust diagnosis and remote monitoring. RF sensing can potentially enable real-time monitoring, data acquisition and analysis, which is valuable in monitoring changes in cognitive function. Unlike conventional medical technologies, RF sensing can offer greater flexibility in terms of user mobility and device placement. This is particularly convenient for applications in care home setups where the patient’s movement needs to be unrestricted during brain monitoring. A framework for remote brain sensing and neurodiagnostics with wearable RF sensing is presented in Figure 1.

In recent years, RF sensing applications have been presented that utilized RF sensors for the detection and imaging of degenerative brain conditions including stroke, brain atrophy, brain tumour, Alzheimer’s disease and dementia. Most of these RF sensing technologies operate in the ultra-wideband (UWB) frequency range, between 1 and 4 GHz. The UWB frequency band offers higher resistance to interference as compared to millimetre waves. This allows deeper penetration within the skin tissues, skull and inner layers of the brain. RF-based sensing techniques operate based on differences in backscattered signal power from the diseased brain tissues. The relative permittivity of the diseased tissues differs from the healthy brain tissues due to differences in composition. The dielectric permittivity in the pathological tissues varies due to disrupted cellular architecture, cell density and ion concentration. Some degenerative pathologies also affect the water content, proteins, electrolytes, ventricular size and blood flow within the brain, which results in the variation in dielectric permittivity. The RF sensors capture these dielectric permittivity changes through reflected or backscattered RF signals. This reflection data can be utilized to reconstruct two-dimensional (2D) images for the visualization of the brain structure and neurodegeneration.

The RF sensing techniques available in the literature are mostly focused on detection and imaging using ultra-wideband sensor arrays. Although the array configuration allows a 360-degree coverage of the brain, power optimization, RF exposure and portable wearability are a few of the challenges in these techniques. RF sensing techniques have been developed for detecting brain atrophy and lateral ventricle enlargement. These studies investigated the dielectric properties of cerebrospinal fluid, which accumulates in the brain cavities and is a major hallmark of Alzheimer’s disease [12]. A Vivaldi antenna was proposed to detect stroke using UWB frequency operation [8]. The array configuration required a switching system for connection and switchover between elements of the antenna array. This required the system to be fixed, and there were mobility limitations. A textile-based antenna was presented for brain imaging, with an operational frequency between 1 and 6 GHz [19]. Due to a relatively larger antenna size, the sensing and imaging system was not suitable to operate in an array configuration. Flexible 16- and 24-element antenna arrays were presented for stroke detection [20,21]. These UWB devices consisted of single-element antennas integrated within a multilayered cap. Although these devices were able to detect stroke within an artificial brain phantom, the device has limitations in terms of wearability and user adaptability. In another study, a wearable RF sensing cap was proposed for brain atrophy detection [11]. A brain atrophy target was emulated on a realistic brain phantom with the placement of cerebrospinal fluid within the brain shrinkage areas. This device was able to detect lateral ventricle enlargement and brain atrophy within the grey and white matter of the brain. An ultra-wideband horn antenna array was presented for haemorrhagic stroke detection, with eight horn antennas placed on a rotatable platform [22]. Although the antenna array was able to detect haemorrhagic stroke and lesions, the system is not portable and supports only static operation with the equipment.

To overcome the limitations of portability, adaptability and larger antenna arrays, this work presents crescent sensors for wearable, portable and non-invasive brain sensing. Crescent sensors provide high bandwidth, improved directivity and enhanced coverage that allow a 360-degree scan of the brain with fewer sensors. The minimalistic crescent sensor design offers easy integration with portable glasses in contrast to the larger Vivaldi and horn antennas. The integration of a portable vector network analyser (VNA) allows the dynamic operation of the sensing device. The multimodal detection capability of the crescent allows the detection of multiple brain anomalies with the same sensors. The remote connectivity via a home gateway and host PC allows the collected backscattered signals to be analysed remotely for 2D imaging and diagnosis with machine learning (ML) models. The portability and remote operability allow the system to be utilized for hybrid diagnostic and imaging with the ML-enabled classification of brain pathology.

## 3. Single Element Crescent Sensors

Single-element crescent sensors are designed to operate on an ultra-wideband (UWB) frequency band. The UWB frequency ranging from 1 GHz to 4 GHz allows the sensors to scan deeper inside the brain to detect neurodegenerative pathologies [23]. The proposed crescent sensors are miniaturized for wearable neurodiagnostic applications. The sensors are designed, simulated and analysed using CST Microwave Studio.

### 3.1. Design Evolution

**Figure 2:**
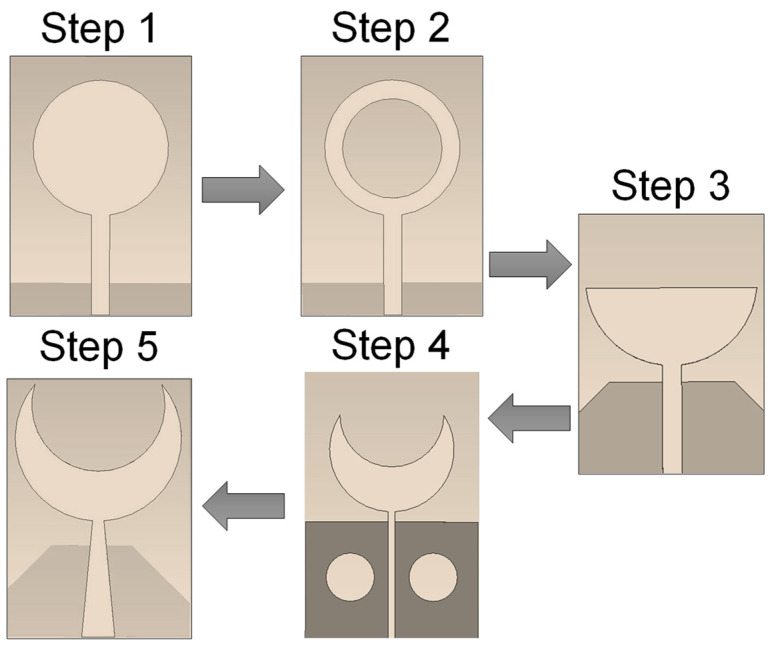
Design stages for single-element crescent sensors.

A circular monopole patch antenna is initially designed on an FR4 substrate with a dielectric constant of 4.4. The design is evaluated on varying substrate thicknesses between 0.8 mm and 1.6 mm. The optimal performance is achieved on a substrate thickness of 1.6 mm. The circular monopole antenna has the dimensions of 60 × 90 mm^2^ and is fed with a microstrip feed line. A partial ground plane is designed to improve impedance matching. Although the circular antenna provided a symmetrical radiation pattern, the S_11_ performance was not optimal, and the overall bandwidth was around 0.8 GHz. To improve performance, a circular slot was introduced in the patch to modify the shape of the radiating element into a donut-shaped circular ring patch. This resulted in an improvement of bandwidth to 1.2 GHz, and the antenna provided a circular polarization. The inner radius of the circular ring is 15 mm, and the outer radius is 18 mm, as shown in Figure 2 (Step 2). The design of the radiating element is then modified to a semi-circular ring, which helps to enhance the bandwidth. The ground structure height is increased with chamfered edges to improve the reflection of UWB frequencies. The design stages along with the final sensor designs are presented in Figure 2.

**Figure 3:**
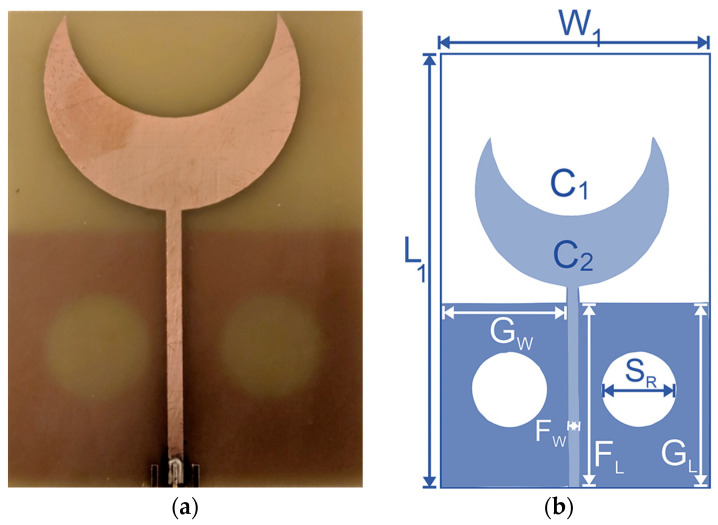
Sensor-1: single-element crescent sensor with circular slots on a partial ground structure. (**a**) Fabricated sensor. (**b**) Geometric configuration.

To achieve a wider bandwidth with improved S_11_ performance, the semi-circular patch structure is modified to a crescent shape by shifting the inner circular slot, as shown in Figure 3. The ground structure is also modified with an increased height, and circular slots are introduced to improve the reflection (S_11_) of the lower frequencies and improve impedance matching. The crescent shape provided a higher bandwidth of around 2 GHz, which ensures the effectiveness of the sensor to operate in near-field neurodiagnostic sensing. Crescent sensor-1 has overall dimensions of 50 × 80 mm^2^ and is designed on an FR4 substrate with a thickness of 1.6 mm and a dielectric constant of 4.4. The radiating element is fed with a 50 Ω feed line, having a width of 2 mm.

**Figure 4:**
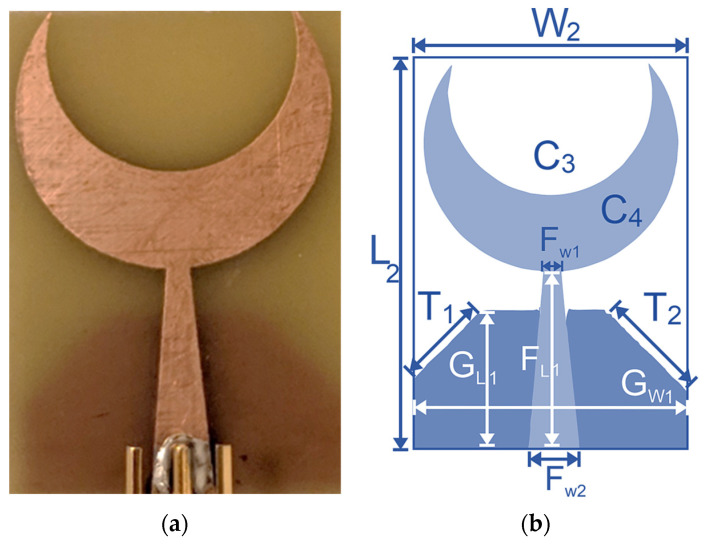
Sensor-2: single-element crescent sensor with tapered feed line and partial ground plane structure. (**a**) Fabricated sensor. (**b**) Geometric configuration.

Sensor-2 is designed with modifications in both radiating elements and ground structure. The design of crescent sensor-2 is further miniaturized by using a tapered feed line and partially chamfered ground structure, as shown in Figure 4. The tapered feed line improves the impedance matching and a partial ground structure results in an improved gain and radiation. There is an overall 40% reduction in the size of the sensor structure, as compared to sensor 1. However, the miniaturization resulted in a bandwidth reduction to 1 GHz. Crescent sensor-2 has overall dimensions of 22 × 31 mm^2^ and is designed on an FR4 substrate with a thickness of 1.6 mm and a dielectric constant of 4.4. The inner circle in this crescent structure has a radius of 8 mm and the outer radius is 10 mm. Other dimensions of the single-element sensors are presented in Table 1.

### 3.2. Reflection Characteristics

Simulations of the designed sensors are carried out using the CST Microwave Studio Suite. The performance of the sensors is computationally verified to assess the bandwidth and operational frequency. As the sensors are designed to operate on ultra-wideband (UWB) frequency for neurodiagnostic applications, the analysis of reflection features is important to assess the performance of sensors. The reflection characteristics are significant as they provide the measure of the antenna matching with its feed line, which indicates a better match for efficient power transfer to the antenna. Moreover, the reflection characteristics influence the radiation pattern of the antenna, and any mismatch due to design constraints may impact the directional characteristics that limit the coverage. This mismatch also affects the sensitivity and detection capability of the sensor. Therefore, careful design is important and enables the sensor to detect weak signals in case of degenerative neuropathology.

**Figure 5:**
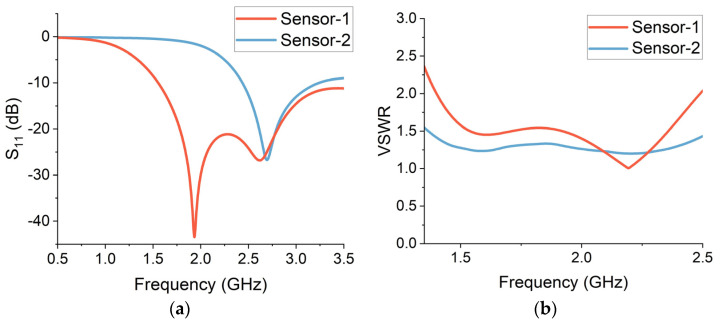
(**a**) Reflection (S_11_) measurements from free-space simulations. (**b**) Simulated voltage standing wave ratio (VSWR) for sensor-1 and sensor-2.

The initial computational analysis is performed using free-space simulations. The reflection (S_11_) measurements were taken through both sensors 1 and 2. The results are presented in Figure 5a. The return loss value is required to be less than −10 dB for the sensor to perform effectively. A return loss of −10 dB corresponds to a reflection coefficient of 0.1. This implies that 90% of the incident power is being transferred to the antenna and only 10% is being reflected. This requires the designed sensor to be well-matched, which also helps to prevent power loss and standing wave effects.

Sensor-1 provides a wide bandwidth of more than 2 GHz ranging from 1.5 GHz to 3.5 GHz. The centre operational frequency is around 1.9 GHz, where the return loss is −43 dB. This indicates that the sensor-1 design is well-matched and is capable of providing high sensitivity for wearable neurodiagnostic applications. Sensor-2 offers an operational bandwidth of around 1 GHz between 2.5 GHz and 3.5 GHz frequencies. The return loss is less than −10 dB over this range, and the return loss value is −28 dB over the centre frequency of 2.6 GHz. Although this bandwidth is comparatively narrower as compared to sensor-1, sensor-2 is more compact and miniaturized.

### 3.3. Gain and Voltage Standing Wave Ratio (VSWR)

The antenna gain refers to the ability of an antenna to direct electromagnetic (EM) signals in a particular direction [24]. The effectiveness of an antenna in the conversion of the input power to radiated EM waves is measured through gain, which is expressed in terms of decibels (dB). The gain is associated with the directivity of the antenna and a higher gain represents high directivity. The design of the antenna is critical in ensuring high gain for wearable applications. As the higher gain requires the antenna to be larger in dimensions, the challenge is to maintain optimal gain performance with a reduced antenna size to ensure a minimalistic device. The simulated gain values for sensor-1 and sensor-2 are 5.3 dBi and 4.9 dBi, respectively.

Voltage standing wave ratio (VSWR) provides a measure of the reflected power on a transmission line. VSWR quantifies the impedance matching level of a transmission line to the connected system [25]. It also offers a measure of efficiency and specifies the effect of standing waves along a transmission line. A higher value of VSWR indicates a high mismatch; however, a VSWR of one indicates that all the power is effectively transferred to the antenna. The realistic threshold of VSWR for radio frequency (RF) sensors is a value that is less than two. This threshold signifies that 90% of power is delivered to the antenna, while 10% is reflected due to impedance mismatch. For wearable sensors, the VSWR is a key parameter that helps to rule out any inefficiencies in the power transfer that are vital to maintain the required antenna sensitivity and coverage. The VSWR results for sensor-1 and sensor-2 are presented in Figure 5b. It can be observed that the sensors provide a VSWR of less than two in their entire operational frequency range. This implies that the sensors can operate effectively over the desired frequencies for neurodiagnostic applications.

### 3.4. Radiation Performance

**Figure 6:**
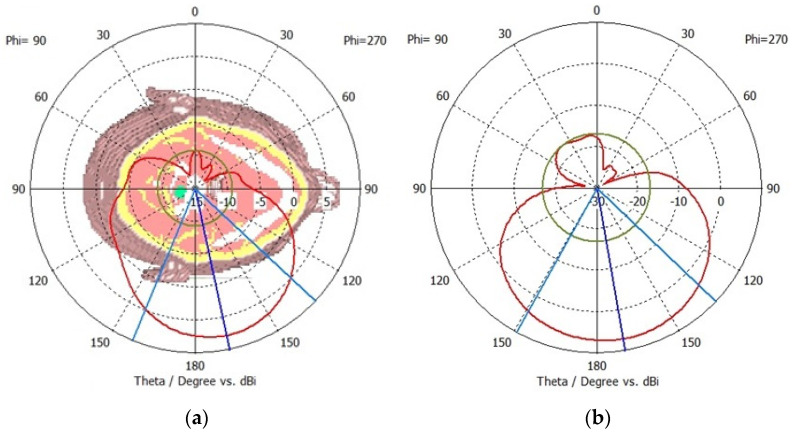
Simulated far-field radiation patterns for (**a**) sensor-1 at 1.9 GHz and (**b**) sensor-2 at 2.6 GHz.

The far-field radiation patterns are simulated and analysed to ensure that the sensors radiate the accepted power in the forward direction. The far-field radiation pattern also validates the antenna’s directivity and capability to suppress radiations in the backwards direction. The far-field radiation pattern is relatively independent of the distance between the antenna and the measurement point, which allows consistent and standardized measurements as compared to the near-field radiation patterns. The radiation patterns are measured on the frequencies of 1.9 GHz for sensor-1 and 2.6 GHz for sensor-2. The results are presented in Figure 6a and Figure 6b for sensor-1 and sensor-2, respectively. Both sensors provide a unidirectional radiation pattern that is suitable for neurodiagnostic applications. The sensors have negligible cross-polarization, and the peak front-to-back ratio is around 18 dB for sensor-1 and 26 dB for sensor-2. A higher front-to-back ratio indicates better directivity and is desirable for neurodiagnostics. Sensor-2 provides a higher front-to-back ratio and, therefore, would be more suitable to suppress interference from unwanted direction. This implies that the sensor is capable of minimizing radiations in the backwards direction.

## 4. Multi-Element Crescent Sensors

Although single-element sensors can detect neurodegenerative pathologies, the arrangement for a complete brain scan may require the placement of multiple sensors around the head. To overcome this, multi-element arrays have been designed that can provide an improved gain and directivity. The design of a sensor array requires the miniaturization and integration of multiple single-element sensors in a single array. For wearable neurodiagnostics, sensor arrays can ensure high sensitivity, miniaturized devices, scalability and low power consumption. The dual- and quad-element crescent sensors presented in this work are fed using a corporate feed. The corporate feed allows the power to be equally split at each junction of the sensor array for a uniform distribution. This configuration provides an enhanced gain by constructively adding the signals from multiple sensor elements and improves the signal-to-noise ratio. This technique also helps in minimizing cross-polarization by directing a major portion of the transmitted power in the desired polarization. This enables deeper penetration within the brain to monitor intricate neurodegenerations. The single-feed arrangement offers a simpler implementation with the least complexity in terms of design, maintenance and data analysis. The single-element crescent design is transformed to form dual- and quad-element crescent arrays.

**Figure 7:**
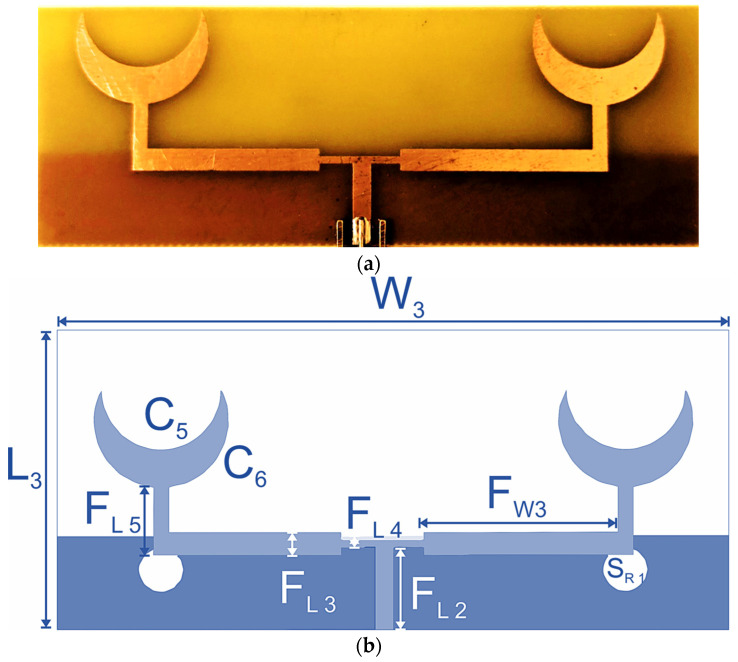
Sensor-3: dual-element crescent sensor. (**a**) Fabricated sensor. (**b**) Geometric configuration.

The dual-element sensor array is designed on an FR4 substrate with a dielectric constant of 4.4 and a thickness of 1.6 mm. The primary feed is designed with a 50 Ω microstrip feed line, having a width of 2.5 mm. The corporate feed network extends through a thin stub of width 0.5 mm and 10 mm in length, which connects the primary feed with the secondary feed line of each element. The extended feed lines that terminate at the T-shaped primary feed junction are 3 mm wide. The parameters for these extended feed lines are varied to achieve the desired length that provides an improved bandwidth. The radiating elements are connected with these extended feed lines through 2.5 mm wide stubs. The fabricated sensor and geometry are presented in Figure 7. The crescent-shaped radiating elements for this dual-element (sensor-3) are comparatively larger than sensor-1 and sensor-2. The overall dimensions of the dual-element sensor are 40 × 90 mm^2^, and the operating frequency is between 2.2 and 3.2 GHz.

**Figure 8:**
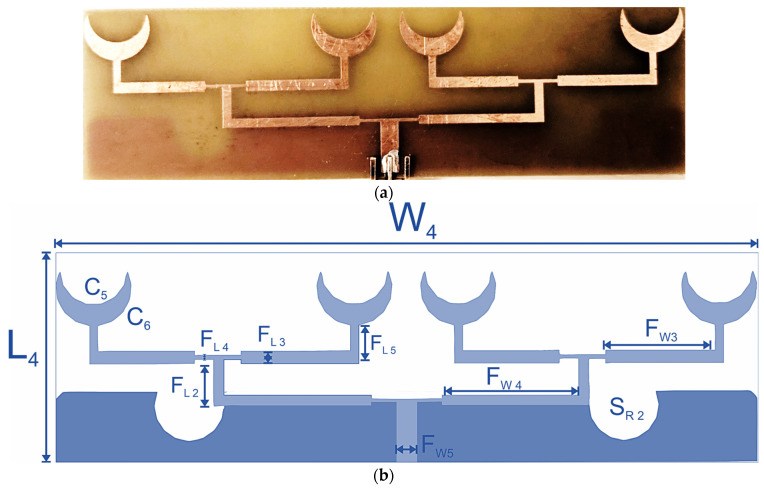
Sensor-4: quad-element crescent sensor array. (**a**) Fabricated sensor. (**b**) Geometric configuration.

The quad-element sensor array is designed with the replication of two dual-element sensors parallel to each other, fed with a corporate feed line. The quad-element array (sensor-4) is designed on FR4 with a thickness of 1.6 mm and a dielectric constant of 4.4. The overall dimensions of the sensor array are 30 × 100 mm^2^. The fabricated quad-element sensor and geometry are presented in Figure 8. The width of the primary feed line is 3 mm and is calculated using the following [26]:

(1) $$\frac{W}{L}={\left[\frac{e^{f}}{8}-\frac{1}{4e^{f}}\right]}^{-1}$$

where *W* is the width and *L* is the length of the sensor array. f is the width of the primary feedline, which can be calculated using the following:

(2) $$f=\frac{Z_{o}\sqrt{2\left(\varepsilon_{r}+1\right)}}{120}+\frac{1}{2}\left(\frac{\varepsilon_{r}-1}{\varepsilon_{r}+1}\right)\left(\ln\frac{\pi }{2}+\frac{1}{\varepsilon_{r}}\ln\frac{4}{\pi }\right)$$

The primary feed is extended to form a T-shaped junction, with a perpendicular stub of length 10 mm. This extends to each pair of crescent arrays via another secondary feed line, which is 3 mm wide and 21 mm in length on each side. This extended secondary feed network is similar to the dual-element sensor in design and dimensions. The radiating elements are designed with an inner radius of 7 mm and an outer radius of 9 mm. Other dimensions of the dual-element and quad-element sensors are presented in Table 2.

### 4.1. Reflection Characteristics

**Figure 9:**
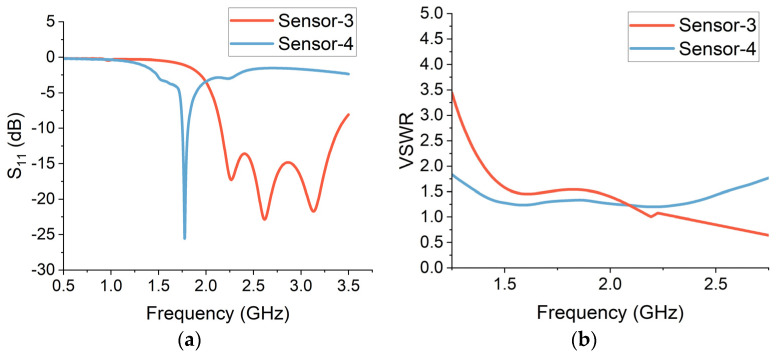
(**a**) Reflection (S_11_) measurements from free-space simulations. (**b**) Simulated voltage standing wave ratio (VSWR) for sensor-3 and sensor-4.

The reflection features of a sensor array represent the individual sensor elements as well as the overall geometry of the array. The reflection patterns of a sensor array can be optimized through antenna geometry, arrangement, number of radiating elements, impedance matching and the spacing between the sensor elements. The performance of sensor arrays can be improved by optimizing these factors. The reflection characteristics of the dual-element and quad-element array sensors are analysed through free-space simulations. The reflection (S_11_) measurements are presented in Figure 9a. The dual-element crescent array (sensor-3) provides a bandwidth of around 1.5 GHz, stretching from 2 GHz to 3.5 GHz. The average return loss is around −20 dB, centred at the frequency of 2.6 GHz. The quad-element crescent array (sensor-4) provides a narrower bandwidth with an operational frequency centred at 1.75 GHz.

### 4.2. Gain and Voltage Standing Wave Ratio (VSWR)

The gain of a multi-element sensor is a measure of its ability to direct the transmitted or received signal in a particular direction when compared to an isotropic radiator. For a multi-element sensor, the gain is influenced by the number of elements, sensor geometry and efficiency. The gain is usually enhanced by increasing the number of elements, as the additional element contributes to the sensor’s ability to focus electromagnetic energy in a particular direction. The sensor geometry and arrangement also affect the gain characteristics. An optimized sensor design is important to realize high gain for wearable applications. To ensure a minimalistic device, it is challenging to maintain optimal gain performance with a smaller sensor size because the higher gain necessitates a larger sensor. The efficiency of each element in a multi-element array also impacts the gain as the inefficient elements in the feed system can reduce the overall effective gain of the sensor. The simulated gain values for sensor-3 and sensor-4 are 5.6 dBi and 6.4 dBi, respectively.

The transmission line’s impedance matching level to the linked system is measured by VSWR. A VSWR of one indicates that all of the power is successfully transferred to the sensor, whereas a higher value denotes a high mismatch. For radio frequency (RF) sensors, a VSWR threshold of less than two is practical. The VSWR results for sensor-3 and sensor-4 are presented in Figure 9b. The VSWR of both multi-element sensors is less than two in their entire operational frequency range. This suggests that the sensors can function efficiently over the desired frequency range for brain-sensing applications.

### 4.3. Radiation Performance

**Figure 10:**
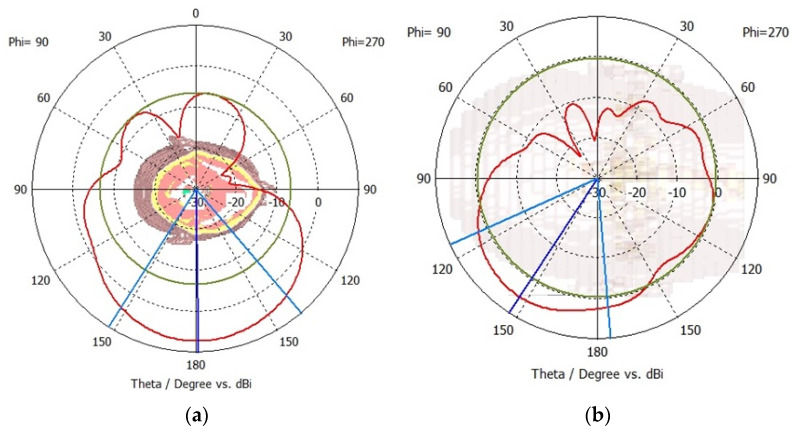
Simulated far-field radiation patterns for (**a**) sensor-3 at 2.6 GHz and (**b**) sensor-4 at 1.75 GHz.

The far-field radiation performance is analysed to ascertain the improvement in multi-element crescent sensors compared to the radiation patterns of single-element sensors. The radiation patterns are measured on the central frequency of 2.6 GHz for sensor-3 and 1.75 GHz for sensor-4. The results are presented in Figure 10a and Figure 10b for sensor-3 and sensor-4, respectively. Both multi-element sensors provide a unidirectional radiation pattern, and the sensors have minimal cross-polarization. The peak front-to-back ratio is 23 dB for sensor-3 and 28 dB for sensor-4. A higher front-to-back ratio is indicative of improved directivity, which is advantageous for brain-sensing applications. This indicates that the multi-element sensors can suppress radiations in the backward direction.

## 5. Flexible Slotted Crescent Sensor

**Figure 11:**
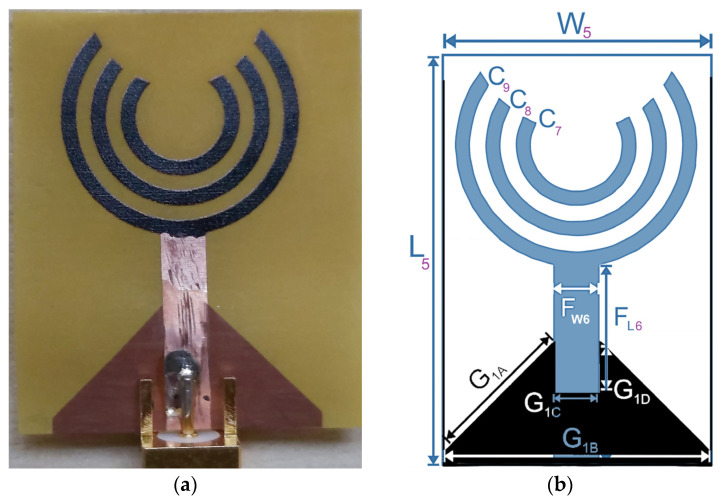
Sensor-5: flexible slotted crescent sensor. (**a**) Fabricated sensor. (**b**) Geometric configuration.

The flexible slotted crescent sensor is designed to operate in the ultra-wideband (UWB) frequency region (1 to 3.3 GHz), which allows the radiofrequency waves to penetrate the brain tissues. The sensor is designed on a flexible polyimide substrate with a thickness of 0.025 mm, loss tangent (tanδ) of 0.0027 and dielectric constant (ε_r_) of 3.5. The proposed sensor is compact with overall dimensions of 18 × 27 mm^2^. The radiating element is a crescent-shaped structure with slotted semi-circular rings in between to optimize the resonant frequency. The flexible sensor has a triangular slotted ground plane, which helps to improve the gain and radiation characteristics. To achieve a wide bandwidth, the sensor design is optimized using narrow slots and wide radiating semi-circular elements within the patch. A partial ground plane is implemented in the final design to ensure wideband performance on lower frequencies, as presented in Figure 11. The dimensions of the initial and final sensor design are presented in Table 3.

### 5.1. Reflection Characteristics

**Figure 12:**
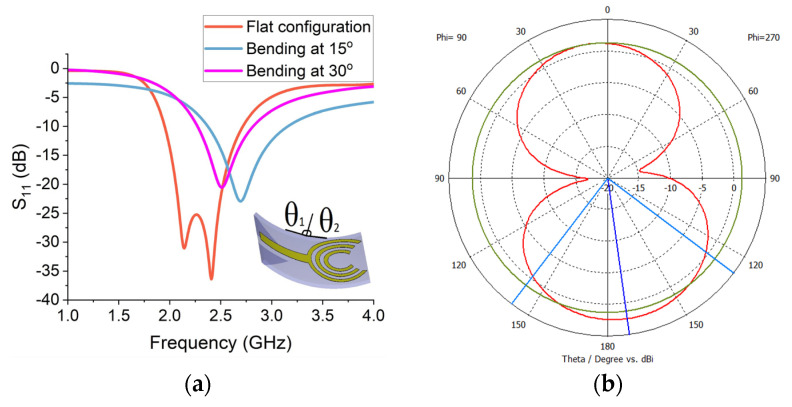
(**a**) Free-space reflection (S11) measurements with flat and bending configurations. (**b**) Simulated far-field radiation pattern for sensor-5 at 2.25 GHz.

The performance of flexible sensors is analysed through free-space simulations. The reflection (S_11_) measurements were taken in both flat and bending configurations to ascertain the effectiveness of this flexible sensor in wearable applications. The S_11_ results are presented in Figure 12a. The flexible slotted crescent (sensor-5) provides a bandwidth of around 1 GHz in a flat configuration, ranging from 1.8 GHz to 2.8 GHz. The peak return loss is around −40 dB at the frequency of 2.4 GHz. The average return loss is around −30 dB over the operational frequency band. This implies that the sensor is highly sensitive and suitable for neurodiagnostics. The performance is further verified by bending the sensor from both sides vertically at θ_1_ and θ_2_, as shown in Figure 12a. The performance is analysed by bending at two angles of 15° and 30° and obtaining the reflection measurements. Although the return loss is slightly reduced and the frequency is shifted forward in both cases, the bandwidth is substantially improved with an increase in the bend.

### 5.2. Gain and Voltage Standing Wave Ratio (VSWR)

The gain of a flexible sensor is normally affected by its compact geometry, flexibility, substrate material and electrical properties. Similar to the single- and multi-element crescent sensors, the operating frequency band also affects the gain. The simulated gain for the flexible crescent (sensor-5) is 4.7 dBi.

The VSWR of the flexible crescent sensor is less than two over the complete ultra-wideband frequency range. The VSWR is verified at a bending angle of 30°, and the value remains under the threshold of 2. This suggests that the flexible sensor can perform effectively in both flat and bending conditions for brain-sensing applications.

### 5.3. Radiation Performance

The radiation patterns are measured on the central frequency of 2.25 GHz for the flexible crescent sensor. The peak front-to-back ratio is 12 dB and the far-field radiation pattern for sensor-5 is presented in Figure 12b. The flexible sensor radiates equally in all directions with the exclusion of two nulls on the phi = 90 plane.

## 6. Near-Brain Simulations

**Figure 13:**
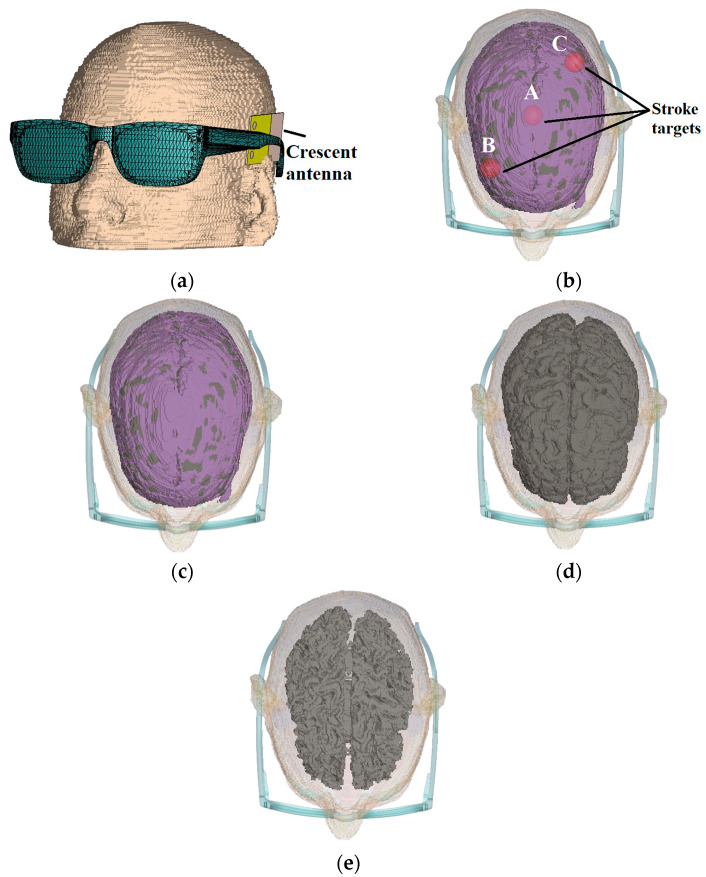
(**a**) Simulation head voxel model with sensor placement on glasses. (**b**) Customized voxel with the placement of stroke target at points A, B and C. (**c**) Normal brain voxel without atrophy and shrinkage. (**d**) Customized brain model representing 15% atrophy of grey matter. (**e**) Brain model with 35% atrophy of grey matter of the brain.

The performance of the designed crescent sensors is verified through near-field analysis. The software simulations are performed on CST Microwave Studio (2022 edition) with a customized brain voxel model constituted of entities like skin, tissues, blood, grey matter, white matter, dura and cerebrospinal fluid. The sensors are mounted on a glasses model and placed next to a realistic brain voxel model, as shown in Figure 13a. The software simulation requires careful dielectric modelling of biological tissues to capture the electrical and biochemical properties of the brain. Therefore, the brain voxel model utilized is customized to incorporate detailed biophysical features. The customized voxel is extracted from MRI images of a real human brain with an improved voxel set [27]. The computational complexity is optimized through adaptive mesh refinement. As the study involved several simulation scenarios with multiple sensors, the simulation complexity is minimized using different mesh sizes to find an optimal balance between simulation accuracy and computational time. The initial measurements were taken with a healthy brain voxel without any changes made to any segment of the brain, as shown in Figure 13c. The stroke target is designed with a composition and relative permittivity similar to the blood but with a high density and high viscosity material [28]. The stroke target is initially placed in the middle of the brain voxel. The placement of the target in the centre of the brain offers an optimal evaluation of designed sensors, as this necessitates the UWB signals to penetrate through the skin, skull, dura and white and grey matter. The performance of sensors is evaluated with the placement of stroke targets at different locations A, B and C within the brain, as shown in Figure 13b.

The relative permittivity of white and grey matter was reduced in the case of brain atrophy [14]. Radiofrequency sensing operates based on dielectric contrast between healthy and diseased tissues. Brain atrophy is generated by reducing the volume of grey matter by 15% initially, with a gradual increase to 35%, as shown in Figure 13d and Figure 13e, respectively. The baseline measurements were recorded with a normal brain voxel. The properties of brain entities within the voxel model are modified to emulate stroke and brain atrophy. The dielectric permittivity (ε), density (kg/m^3^) and conductivity (S/m) of blood, cerebrospinal fluid (CSF) and white and grey matter are altered, as presented in Table 4. The reflection measurements were taken after each alteration and compared to the baseline measurements of a healthy brain. In the case of brain atrophy, the permittivity of both white and grey matter generally decreases due to the loss of neurons and overall cellular structure. As a result, the density of white and grey matter also decreases. Conversely, the conductivity of CSF increases due to an increase in CSF volume within the ventricles and around the brain. Although brain tissues have lower conductivity than CSF, the shrinkage of tissues effectively allows for electrolytes to be present in a larger volume, potentially leading to higher conductivity.

**Figure 14:**
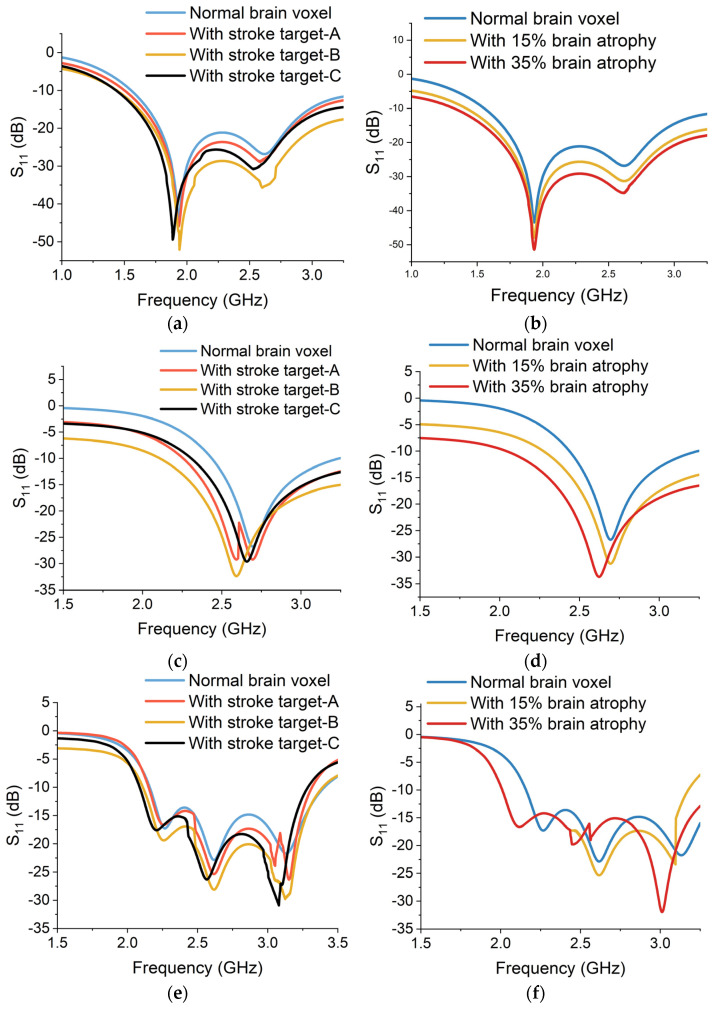

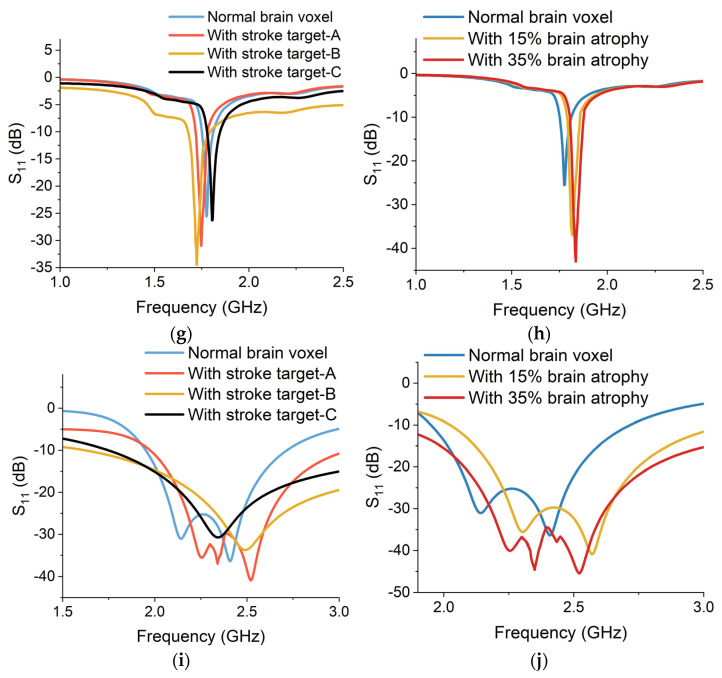
Simulated reflection (S_11_) results from sensor-1 for (**a**) brain stroke and (**b**) brain atrophy, sensor-2 for (**c**) brain stroke and (**d**) brain atrophy, sensor-3 for (**e**) brain stroke and (**f**) brain atrophy, sensor-4 for (**g**) brain stroke and (**h**) brain atrophy and sensor-5 for (**i**) brain stroke and (**j**) brain atrophy.

The simulated reflection S_11_ measurements are obtained from each sensor to assess their performance in detecting stroke and atrophy degeneration. The simulation results are presented in Figure 14. In the case of a stroke target, the backscattered power is relatively lower than the power from a normal voxel. The electromagnetic properties of stroke-affected regions differ from the normal brain tissues due to the presence of oedema, blood clots and damaged cells. Therefore, the UWB signals reflect differently from the stroke-affected targets, as can be seen in Figure 14a,c,e,g,i. In addition, the location of the stroke target within the brain affects the S_11_ measurements. The average backscattered signal power is lower in the case of stroke target A, as compared to targets B and C. Stroke target B is placed on the front of the brain and is relatively closer to the placement of sensors. Therefore, the reflected power received at the sensors from target B is higher in contrast to targets A and C. This implies that the proximity of the sensor to the stroke location can influence the accuracy and sensitivity of S_11_ measurements.

Similarly, for brain atrophy, the reflected power decreases as the brain shrinkage and atrophy increase. The power is being absorbed or transmitted through the atrophy regions and less power is being reflected towards the sensors. The CSF accumulation reduces the dielectric permittivity of the atrophy region. Therefore, the level of backscattering is comparatively higher in the case of 35% atrophy than in 15% atrophy simulation, as shown in Figure 14b,d,f,h,j.

### 6.1. Specific Absorption Rate

**Figure 15:**
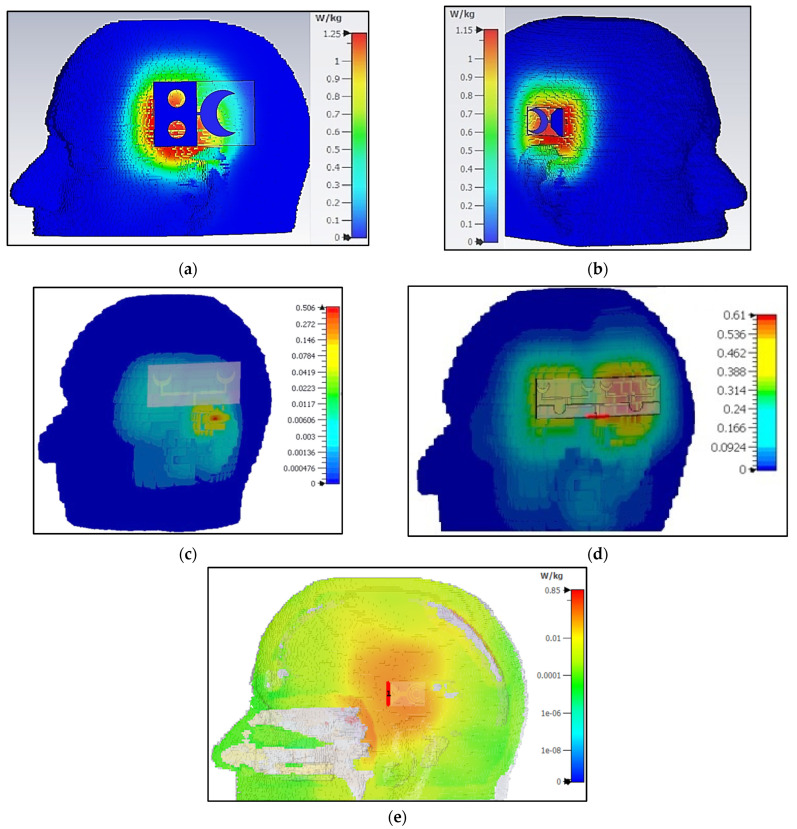
Simulated specific absorption rate (SAR) for (**a**) sensor-1 at 1.9 GHz, (**b**) sensor-2 at 2.6 GHz, (**c**) sensor-3 at 2.6 GHz, (**d**) sensor-4 at 1.75 GHz and (**e**) sensor-5 at 2.25 GHz.

Specific absorption rate (SAR) is a key parameter to quantify the energy absorbed by the biological tissues when exposed to electromagnetic fields generated by the wearable sensors. SAR is used as a safety guideline in the design and evaluation of wearable communication and sensing devices to ensure that the exposure to RF signals does not exceed the standard safety limits [29]. Regulatory bodies, such as the International Commission on Non-Ionizing Radiation Protection (ICNIRP) and the Federal Communications Commission (FCC), have established SAR limits to restrict RF exposure. The ICNIRP standard allows a maximum of 2 W/Kg SAR measured on 10 g of tissue mass [30]. To validate the SARs of the proposed sensors, near-field analysis is performed in CST Microwave Studio to calculate SAR using a brain voxel model. The SAR results are presented in Figure 15 and are calculated against the input power of 100 mW. The SARs of all sensors is within the prescribed limit, with sensor-1 having a maximum SAR of 1.25 W/Kg, while sensor-3 has a minimal SAR of 0.50 W/Kg for an input power of 100 mW.

### 6.2. Thermal Characterization

Thermal analysis involves simulating the electromagnetic fields and power absorption within the brain tissues. As the wearable sensors are designed to operate in the near-field, the electromagnetic waves can increase tissue temperature. A higher specific absorption rate (SAR) implies more power absorption within the tissues. The power absorption in each voxel tissue can be determined using the electromagnetic field distribution, tissue properties and the SAR. The power absorption by the tissues can be used to determine the average temperature rise within the tissues [31]. Other factors that affect this temperature rise include specific heat and thermal conductivity. The thermal effect of crescent sensors on the brain is measured using the CST transient temperature solver and the results are presented in Table 5.

### 6.3. Electric Field Analysis

The computational model is evaluated to measure electric field intensity using field probes placed at various regions of interest within the brain. The electric field probes offer valuable insight into the behaviour of electromagnetic fields in the vicinity of sensors [32]. The electric field probes quantify the strength of the electric field at specific regions within the brain voxel model. The simulations for electric field analysis were conducted by sensor placement at 10 mm, 15 mm and 20 mm spacing to validate the optimized antenna–tissue separation that offers minimal interference. The optimal results are achieved with the sensor placement at 10 mm away from the brain. The proposed crescent sensors are evaluated by placing the electric field probes inside the skull, dura and grey and white matter of the brain. The measured results of the peak electric field (V/m) at these brain regions are presented in Table 6. The results indicate that the electric field strength is reduced at the probes placed deep within the brain, around grey and white matter. This is due to the impact of skin tissues, skull and dura layer on the transmitted electromagnetic waves.

## 7. Experimental Validation

**Figure 16:**
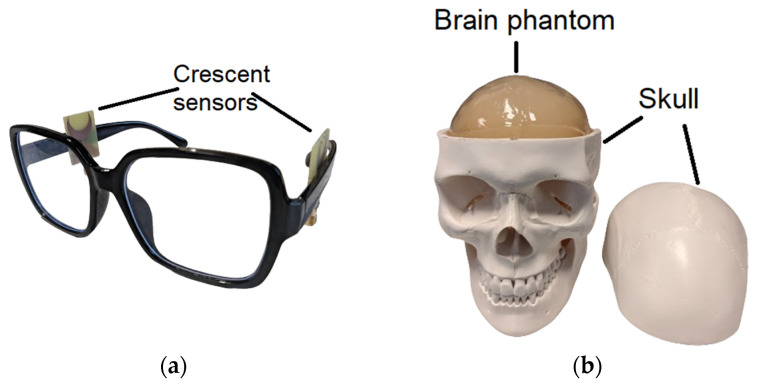
(**a**) Glasses with integrated crescent sensors for experimentation. (**b**) Fabricated gel-based brain phantom customized with stroke and brain atrophy targets.

The proposed crescent sensors have been experimentally verified on artificial brain phantom for multiple neurodegenerative pathologies including stroke and brain atrophy. The proposed sensors detect neurodegeneration on the basis of variations in reflection from the brain. The RF signals are transmitted towards the brain and the dielectric changes in the brain, due to these pathologies, result in the backscattering of signals. The backscattered signals are collected on a host PC through the Vector Network Analyzer (VNA). These backscattered signals are analysed to determine the extent of neurodegeneration. The experimentation is performed on a realistic brain phantom prepared with a mixture of sugar, agar and water. The overall concentration of the phantom material is 1400 mL, which is closely associated with the actual brain volume of a human adult [33]. The dielectric properties of the mixture were measured during the mixing procedure to match it with the real brain [34]. The phantom mixture is placed within an artificial skull, as shown in Figure 16b.

The sensors are experimentally evaluated on neurodegenerations associated with brain stroke and brain atrophy. The initial measurements were taken on a normal phantom with no degeneration. This initial measurement is taken with each sensor as a reference benchmark. Environmental changes can influence the S_11_ measurements and factors like temperature, humidity levels and electromagnetic interference may impact the results. Therefore, to minimize the environmental changes, the results were taken in a controlled lab environment with regulated temperature and humidity conditions. The sensor-specific and measurement artifacts are mitigated through the calibration of VNA ports and SMA cables before each set of measurements. The noise and environmental artifacts are further minimized through an optimal adjustment of antenna–tissue separation. As the sensors are designed for integration with glasses, the configuration helps to avoid motion artifacts.

Brain stroke is emulated using a spherical stroke target placed in the middle of the brain phantom. The volume of the stroke target is 25 mm^3^ and is filled with saline liquid to mimic the dielectric properties of an actual blood accumulation. The single- and multi-element crescent sensors are individually placed around the brain phantom to obtain the measurements. For illustration, the placement of sensor-2 on glasses is presented in Figure 16a.

**Figure 17:**
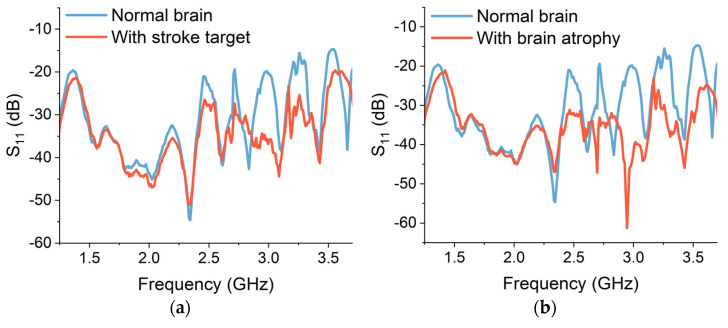

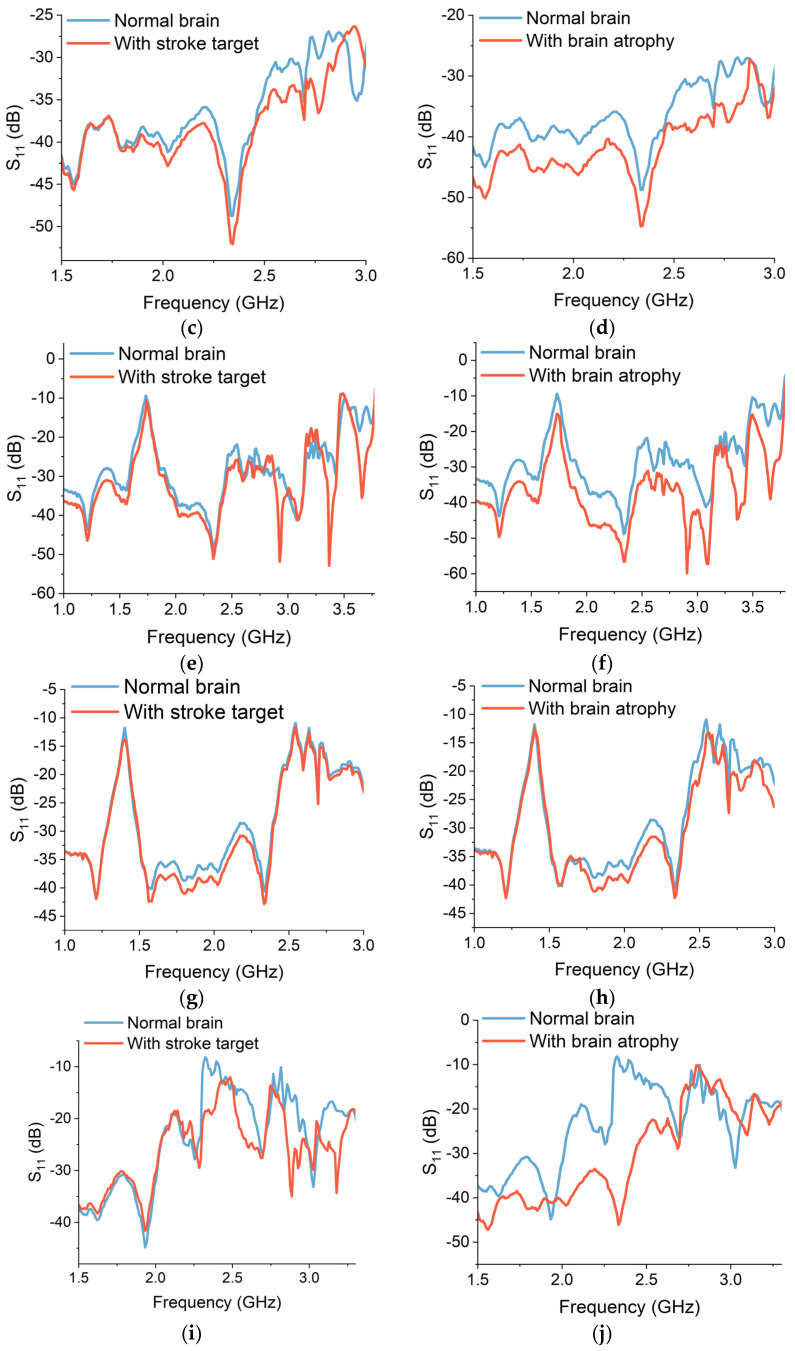
Measured reflection (S_11_) results from sensor-1 for (**a**) brain stroke and (**b**) brain atrophy, sensor-2 for (**c**) brain stroke and (**d**) brain atrophy, sensor-3 for (**e**) brain stroke and (**f**) brain atrophy, sensor-4 for (**g**) brain stroke and (**h**) brain atrophy and sensor-5 for (**i**) brain stroke and (**j**) brain atrophy.

Brain atrophy is emulated using a cavity of 56 mm^3^ volume in the centre of brain matter [11]. The brain atrophy is filled with a mixture of saline liquid and gelatine, which represents the cerebrospinal fluid (CSF). The dielectric properties of this mixture were first measured with a dielectric probe to match the relative permittivity with real CSF. Similar to the stroke analysis, the measurements are taken individually with the placement of single- and multi-element crescent sensors on the glasses. The measured results are taken with stroke and brain atrophy and compared with the baseline measurements, as shown in Figure 17. It can be observed that the magnitude of reflection of S_11_ reduces in the presence of stroke and brain atrophy targets. The reflected power is comparatively lower in the case of brain atrophy, which is due to a larger volume of the atrophy resulting in higher absorption of electromagnetic waves. The measured experimental results are consistent with the simulated results for the detection of stroke and brain atrophy.

The variation in S_11_ magnitude represents the intensity of reflected electromagnetic waves that are received by the sensors. This variation can be characterized in terms of deviation from the mean values to assess the degeneration. These variations are analysed in terms of mean S_11_ and standard deviation in the magnitude S_11_ and presented in Table 7. The results for stroke in Figure 17a,c,e,g,i indicate a smaller change in the reflection from the stroke target, which is due to the lower volume of the target (25 mm^3^). The increase in the volume of brain atrophy (56 mm^3^) increases the S_11_ loss, due to the cavity within the brain and the CSF accumulation within the cavity. This affects the dielectric properties of the atrophy region as CSF is more conductive due to low cellular composition and high water content. The higher downward trend in S_11_ can be observed for the brain atrophy in Figure 17b,d,f,h,j. This validates the sensors’ capability to detect the changes in volume and dielectric properties of the pathological tissues.

## 8. Fabrication Materials and Techniques

A wide range of materials and techniques has been investigated in the recent literature for the fabrication of wearable sensors. The major factors that can impact the performance of the sensors include material biocompatibility, sensitivity, specificity, stability, flexibility and user experience. Wearable RF sensors are primarily composed of substrate materials and the choice of substrate is crucial in ensuring optimal sensor performance. Flexible substrate allows improved comfort for wearability due to their flexibility and structural integrity. Some applications require the integration of sensors with wearable devices, which requires the sensors to be rigid for easy integration and operability.

The wearable sensors reported recently in the literature utilized polymeric materials like polydimethylsiloxane (PDMS), polyurethane (PU), EcoFlex and flexible FR4. These polymer-based materials provide flexible structural and electrical properties, which makes them suitable for wearable applications. Textile materials have also been utilized in respiration and brain sensing as they offer flexibility and durability with ease of use. The paper-based substrates are also reported for wearable sensing applications. The paper-based sensors are cost-effective, easy to manufacture and provide improved biocompatibility [35]. Table 8 summarizes the properties of the substrate materials reported in the literature and the fabrication material utilized in this work.

The substrates utilized in the proposed work to design crescent sensors are FR4 and polyimide. Flame-retardant glass-reinforced epoxy laminate (FR4) is a widely used rigid substrate material due to its cost-effectiveness and better electrical insulation. The selection of the substrate thickness is critical as it affects the sensor impedance and radiation performance. The two-layered FR4 substrate consists of a thin layer of copper on both sides. The selection of FR-4 for the single- and multi-element sensor design is due to their possible integration with the glasses, and the material rigidity is suitable for this. Polyimide substrates are often used to manufacture flexible electronics and printed circuit boards. Polyimide offers greater flexibility, dimensional stability, thermal stability and heat resistance. Polyimide can withstand high temperatures without losing its structural integrity. This resistance makes it suitable for wearable applications and provides high tensile strength and flexibility.

## 9. Comparative Analysis

Radio frequency (RF) sensors have been the focus of research for biomedical sensing applications in recent years. Several studies have been reported for the detection of brain tumours, brain stroke, brain infarction and Alzheimer’s disease. These techniques mostly utilized an array of antennas or bulky antennas, placed around the target to achieve a 360-degree coverage of the brain. The portability, power optimization, RF exposure and array configuration remain a challenge in these sensing techniques. Some of these techniques can only operate in a static configuration, which limits the mobility of the patient. Table 9 presents a comparison of proposed crescent sensors with state-of-the-art wearable sensors. In contrast to the available sensing techniques, the proposed crescent sensors provide optimal performance in terms of sensitivity, directivity, resolution and detection capability with a low profile, conformable and cost-effective design. Crescent sensors offer a minimalistic design in contrast to the bulky, rigid horn, helical and Vivaldi antennas. The integration of sensors with glasses allows wearability, portability and mobility to the user.

**Figure 18:**
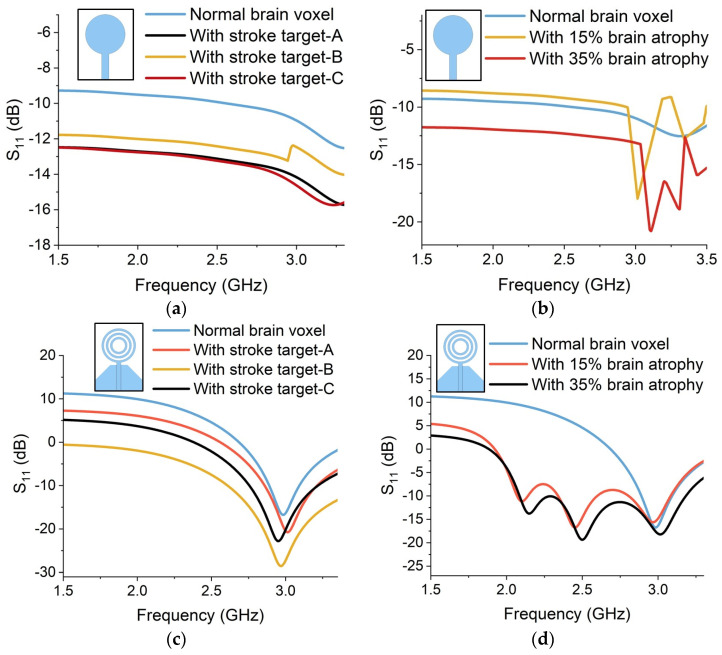
Simulated reflection (S_11_) results from the circular sensor for (**a**) brain stroke and (**b**) brain atrophy and the multi-ring sensor for (**c**) brain stroke and (**d**) brain atrophy.

Crescent sensors provide an optimal performance in comparison to other microstrip designs. The performance of crescent sensors is compared to the circular and multi-ring-shaped sensors for stroke and brain atrophy detection, as shown in Figure 18. The baseline measurements were taken on a normal brain voxel with no anomaly. These simulated results indicate that the circular and multi-ring sensors have a narrow bandwidth with an operational frequency of 3.5 GHz and 3 GHz for circular and multi-ring sensors, respectively. However, the crescent sensors proposed in this work provided a wide bandwidth, as presented in Figure 14a,c,e,g,i. Moreover, the circular and multi-ring sensors are less sensitive to backscattered electromagnetic signals. This leads to less variability in the reflection measurements from stroke and atrophy targets, as shown in Figure 18a,b and Figure 18c,d for circular and multi-ring sensors, respectively.

The reflection results for stroke indicate an average and standard deviation of 13.5 ± 3.2 dB and 19.8 ± 6.6 dB for circular and multi-ring sensors, respectively. Additionally, the reflection statistics for brain atrophy show an average and standard deviation of 14.7 ± 6.2 dB and 15.1 ± 4.8 dB for circular and multi-ring sensors, respectively. Conversely, the proposed crescent sensors provide a maximum average and standard deviation of −39.65 ± 13.49 dB for sensor-2, as shown in Table 7. Based on these statistical results, the crescent sensors provide more variability in the reflection data, which is useful for quantifying the potential variations in the data. This validates the selection of crescent sensors for the proposed sensing device.

## 10. Opportunities and Challenges

Radio frequency (RF) sensors can be effectively utilized in neurodiagnostic applications for monitoring, data acquisition and imaging. The flexibility, non-invasiveness, low power consumption, safety and reduced interference make these sensors viable for wearable applications. The robustness, ease of use and patient comfort enable the RF-sensing devices to be deployed for remote monitoring. The proposed RF-based crescent sensors offer most of these features, making them an ideal choice for a wearable neurodiagnostic tool. The miniaturized single-element crescent sensors can be easily mounted on glasses to achieve complete coverage of the brain. These portable head-mounted devices can be utilized for remote patient monitoring in either clinical or care home settings. These sensors provide optimal performance with low power consumption and ease of operability, in contrast to conventional medical technologies like functional magnetic resonance imaging (fMRI), computed tomography (CT) and ultrasound imaging. The sensing data collected from the crescent sensors can be utilized to reconstruct brain images for the visualization of neurodegeneration. The imaging process can be made more robust with the use of machine learning models, which would help characterize the sensing and imaging data. This will be helpful in robust diagnosis based on reconstructed images for the particular neurodegeneration. The process of data collection, artifacts removal, storage, feature extraction and pattern recognition can be performed with encrypted processing on the cloud. Cloud processing platforms will enable healthcare professionals to accelerate diagnostic workflow with remote access to RF-enabled diagnostic tools. As the RF sensors are designed to operate with low power consumption, they are suitable for continuous portable monitoring. There is a potential to exploit RF sensing for brain activity monitoring, which may contribute to detailed visualization of brain functionality and neurological conditions.

The challenges associated with radio frequency (RF) brain sensing are multifaceted and include physiological and ethical considerations. Achieving accurate brain activity measurements and diagnostics through RF backscattered signals requires overcoming complexities in signal processing. This involves ensuring sufficient depth of penetration to capture activity in deep brain structures and addressing limitations in spatial resolution. The interference and noise in RF backscattered signal data pose challenges in maintaining signal reliability. Therefore, the need for rigorous validation against established brain sensing methods is essential for clinical translation. As this study is conducted on a realistic brain phantom in a controlled environment, the external factors are substantially minimalized. The near-field impact of the human biological tissue and sensor interaction will be studied in future in vivo experiments. These tests will involve pre-clinical trials of the sensors that will account for the complexities of the human brain and the environment. Balancing the non-invasive nature of RF sensing with the preservation of privacy and security raises ethical concerns, necessitating robust measures to protect sensitive brain-related data from unauthorized access. Furthermore, adherence to health and regulatory standards and guidelines is crucial to ensure the safety and ethical use of RF brain-sensing technologies in healthcare and neuro-sensing applications. Another challenge would be to capture the dielectric properties of human tissues that vary due to demographics. To overcome this, an in vivo data set will be collected and segregated into individual classes based on the demographics of human subjects. The baseline measurements will be segregated accordingly into separate classes. Statistical models coupled with machine learning (ML) algorithms will be utilized to train algorithms for improved detection accuracy. The edge computing will enable on-device and distributed learning at the edge device, integrated with glasses, for real-time diagnostics with privacy preservation. This will enable on-device inference with an immediate analysis of tissue properties. The edge-based ML implementation of the wearable sensing device will require careful consideration of ethical guidelines surrounding data privacy and bias mitigation. Overall, the opportunities and prospects outweigh the challenges, and non-invasive RF sensing will potentially revolutionize the neurodiagnostic field in the near future. The capability of RF sensing to cover a broad range of brain regions, the possibility of integration with other imaging modalities and the potential for remote monitoring suggest an impactful application in neurodiagnostics. The possible improvement in signal processing algorithms, combined with ongoing research in RF technology, has the potential to address current challenges and enhance the accuracy and reliability of brain pathology measurements. RF brain sensing can contribute to robust diagnostics, personalized imaging, and therapeutic interventions.

## 11. Conclusions and Future Work

This study presents a comprehensive overview of the design, fabrication and performance analysis of crescent sensors for sensing brain pathology. The crescent sensors offer high conformability, low profile, flexibility, high sensitivity, cost-effectiveness and ease of fabrication in contrast to the horn, helical and Vivaldi antennas. The miniaturized structure allows the crescent sensors to easily integrate with the wearable devices. The crescent sensors are capable of sensing different brain pathologies with real-time robust monitoring. The sensors are integrated with glasses after near-field computational analysis with a software brain voxel model. The sensors can detect different brain atrophy levels and stroke targets placed at varied locations within the brain. The fabricated device is experimentally validated on a realistic brain phantom with pathologies indicative of stroke and brain atrophy. The sensors can successfully detect stroke and brain atrophy targets with a volume of 25 mm^3^ and 56 mm^3^, respectively. The simulated and measured results of the proposed sensors are consistent, which validates the effectiveness of crescent sensors for brain-sensing applications. Future work will focus on the integration of wireless 5G sensors for live data processing and diagnostics. The integration of machine learning models will enable hybrid sensing and imaging for robust classification and medical intervention. The miniaturization, energy efficiency and edge computing can enable on-device and distributed learning at the edge devices for real-time diagnostics with privacy preservation. The multimodal fusion of radio frequency sensors with other wearable vitals sensing modalities can improve diagnostics.

## Figures and Tables

**Table 1:** Dimensions of the single-element crescent sensors.

Parameter	Description	Dimensions (mm)
L_1_	Length of sensor-1	27
W_1_	Width of sensor-1	18
F_W_	Feed line width of sensor-1	3
F_L_	Feed line length of sensor-1	16
C_1_	Circular inner semi-ring radius of sensor-1	3.5
C_2_	Circular outer semi-ring radius of sensor-1	7.5
G_L_	Ground plane height for sensor-1	9
G_W_	Width of the ground plane for sensor-1	18
S_R_	Width of circular slots in the ground plane of sensor-1	3
L_2_	Length, final design of single-element sensor	17
W_2_	Width, final design of single-element sensor	14
F_W1_	Feed line width facing radiating patch, for sensor-2	2
F_w2_	Feed line width facing feeding port, for sensor-2	9
F_L1_	Feed line length of sensor-2	2.5
C_3_	Inner semi-ring radius of sensor-2	4
C_4_	Outer semi-ring radius of sensor-2	5.5
G_L1_	Ground plane height for sensor-2	3.8
G_W1_	Width of the ground plane for sensor-2	6.4
T_1_	Chamfered ground plane length on the left side of sensor-2	5
T_2_	Chamfered ground plane length on the right side of sensor-2	4

**Table 2:** Dimensions of the multi-element crescent sensors.

Parameter	Dimensions (mm)	Parameter	Dimensions (mm)	Parameter	Dimensions (mm)
L_3_	40	W_3_	90	F_W3_	26
F_L2_	11	F_L3_	3	F_L4_	0.5
F_L5_	9	C_5_	7	C_6_	9
S_R1_	3	L_4_	30	W_4_	100
F_W4_	21	F_W5_	3	S_R2_	5

**Table 3:** Dimensions of the flexible slotted crescent sensor.

Parameter	Description	Dimensions (mm)
L_5_	Length of sensor	27
W_5_	Width of sensor	18
F_W6_	Feed line width	3
F_L6_	Feed line length	16
C_7_	Circular inner semi-ring radius	3.5
C_8_	Circular middle semi-ring radius	5.5
C_9_	Circular outer semi-ring radius	7.5
G_1A_	Ground plane chamfer width	9
G_1B_	Width of ground plane	18
G_1C_	Width of rectangular slot in ground	3
G_1D_	Length of rectangular slot in ground	3.5

**Table 4:** Simulation parameters for stroke and brain atrophy.

Brain Voxel Segment	Normal Brain			Stroke			Brain Atrophy					
	ε	ρ	σ	ε	ρ	σ	15% Atrophy			35% Atrophy		
							ε	ρ	σ	ε	ρ	σ
Blood	63.2	1050	1.38	63.2	1050	1.38	63.2	1050	1.38	63.2	1050	1.38
Cerebrospinal fluid	71	1100	1.79	71	1100	1.79	66	1005	2.15	62	1005	2.25
White matter	40.5	1041	0.47	63.2	1041	0.77	36.5	650	0.51	34	545	0.59
Grey matter	54.8	1045	0.77	63.2	1045	0.77	41	825	0.58	37.5	740	0.64
Stroke target	-	-	-	67	1190	1.55	-	-	-	-	-	-

**Table 5:** Thermal effect of crescent sensors on brain (maximum temperature increase in Celsius).

	Skin Tissues	Skull	Dura	Grey Matter	White Matter
Sensor-1	0.155	0.142	0.139	0.127	0.121
Sensor-2	0.145	0.136	0.128	0.119	0.114
Sensor-3	0.161	0.152	0.149	0.142	0.136
Sensor-4	0.173	0.166	0.158	0.149	0.138
Sensor-5	0.134	0.129	0.125	0.121	0.115

**Table 6:** Electric field measurements at different brain regions in near-field operation (V/m).

	Skull	Dura	Grey Matter	White Matter
Sensor-1	4.52	4.39	3.74	3.61
Sensor-2	2.73	2.35	1.85	1.62
Sensor-3	3.54	3.42	3.06	2.89
Sensor-4	3.72	3.63	3.24	2.97
Sensor-5	2.56	2.42	2.11	1.94

**Table 7:** Statistical analysis of measured reflection from stroke and brain atrophy targets.

	Normal Brain		With Stroke Target		With Brain Atrophy	
	Mean (dB)	Standard Deviation (dB)	Mean (dB)	Standard Deviation (dB)	Mean (dB)	Standard Deviation (dB)
Sensor-1	−30.15	±7.91	−33.17	±9.39	−34.85	±11.43
Sensor-2	−34.12	±9.87	−36.32	±11.63	−39.65	±13.49
Sensor-3	−27.15	±9.63	−31.48	±10.62	−36.54	±12.35
Sensor-4	−26.41	±8.49	−30.38	±9.29	−34.14	±11.07
Sensor-5	−25.98	±8.60	−27.42	±9.88	−31.26	±10.95

**Table 8:** Comparison of fabrication material used for wearable sensors.

Substrate Material	Flexibility	Tensile Strength	Conformability	Biocompatibility
Polydimethylsiloxane, PDMS [36]	Yes	Low	High	High
EcoFlex [37]	Yes	Medium	Medium	Medium
Polyurethane [38]	Yes	Medium	Medium	Medium
Textile [7]	Yes	Medium	High	High
Paper [39]	Yes	Low	Medium	Medium
Flame retardant, FR4 (this work)	No	High	Medium	Medium
Polyimide (this work)	Yes	High	High	High

**Table 9:** Comparison of proposed crescent sensors with state-of-the-art wearable sensors.

References	Sensor Type	Dimensions (mm)	Device Type	Number of Antennas in the Device	Operating Frequency (GHz)	Sensor Resolution (Detectable Volume, mm^3^)	Target Application
[22]	Horn	120	Rotatable platform	8	0.5 to 6	40	Haemorrhagic stroke
[8]	Vivaldi	73.4 × 41.9	Rotatable platform	3	4 to 12	-	Brain cancer
[11]	Planar monopole	70 × 30	Wearable (hat)	6	0.8 to 3	22.6 to 226	Brain atrophy
[14]	Stepped monopole	85 × 35	Wearable (hat)	6	1.5 to 3	22.6 to 226	Alzheimer’s disease
[6]	Antenna array	360 × 300	Wearable (standalone)	8	1.16 to 1.94	62.5	Brain stroke
[21]	Planar antenna array	70 × 50	Wearable (cap)	24	0.9 to 2.5	94 to 360	Brain stroke
[20]	Planar multi-slot microstrip	360 × 300	Wearable (cap)	16	0.6 to 2.5	30	Brain stroke
[40]	Square monopole	76 × 38	Wearable (standalone)	8	1.1 to 1.3	30	Haemorrhagic stroke
Sensor-1	Crescent with circular ground slots	27 × 18	Wearable (glasses)	2	1.5 to 3.5	25	Brain stroke and brain atrophy
Sensor-2	Crescent with partial ground plane	17 × 14	Wearable (glasses)	2	2.5 to 3.5	25	Brain stroke and brain atrophy
Sensor-3	Dual-element crescent array	90 × 40	Wearable (glasses)	2	2 to 3.5	25	Brain stroke and brain atrophy
Sensor-4	Quad-element crescent array	100 × 30	Wearable (glasses)	2	1.7 to 1.8	25	Brain stroke and brain atrophy
Sensor-5	Flexible slotted crescent	27 × 18	Wearable (glasses)	2	1.8 to 2.8	25	Brain stroke and brain atrophy

## Data Availability

Data is contained within the article.
